# Supplementary material for: PPAR-Gamma Activation May Inhibit the In Vivo Degeneration of Bioprosthetic Aortic and Aortic Valve Grafts under Diabetic Conditions
Source: Int J Mol Sci. 2021 Oct 14;22(20):11081. doi: 10.3390/ijms222011081 (PMC8538504; doi:10.3390/ijms222011081)
Supplement: Supplementary file 1 [file ijms-22-11081-s001.zip › Fig S4 + caption.pdf]

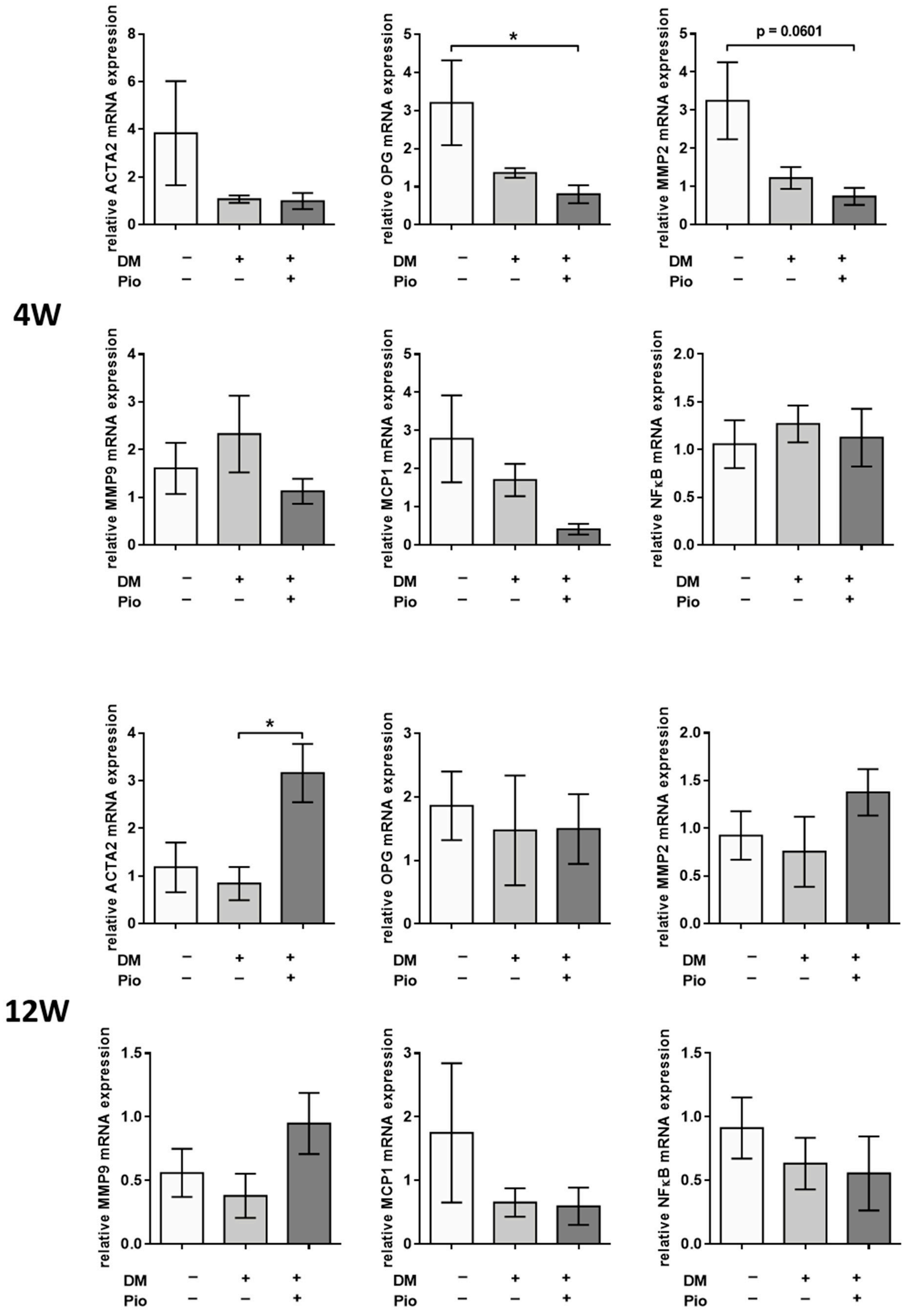

Supplemental Figure S4: Gene expression analysis of targets related to bio-prosthetic valve degeneration. Analysis of gene expression was performed in aortic grafts 4 and 12 weeks after implantation with n = 6 animals for each analysis group. Treatment- and time-dependent expression

profiles were detectable for genes related to cell differentiation (ACTA2) and tissue degeneration (OPG) as well as related to collagen remodeling (MMP2). Markers for inflammation (MCP-1, NFκB) were in contrast not affected by pioglitazon at both time points of explantation. ACTA2, actin alpha 2; OPG, osteoprotegerin; MMP2, matrix metalloproteinase 2; MMP9, matrix metalloproteinase 9; MCP-1, monocyte chemoattractant protein 1; NFκB, nuclear factor kappa-light-chain-enhancer of activated B cells;*, $p<0.05$. Data was analyzed using Kruskal-Wallis with Dunn's multiple comparisons test.
