# Supplementary material for: PPAR-Gamma Activation May Inhibit the In Vivo Degeneration of Bioprosthetic Aortic and Aortic Valve Grafts under Diabetic Conditions
Source: Int J Mol Sci. 2021 Oct 14;22(20):11081. doi: 10.3390/ijms222011081 (PMC8538504; doi:10.3390/ijms222011081)
Supplement: Supplementary file 1 [file ijms-22-11081-s001.zip › Supplemental data_methods.pdf]

Supplemental Methods

Functional heterotopic implantation model

The model involves a staged procedure with the first stage represented by a closed chest intervention, where the native aortic valve of the recipient animal is punctured and native aortic valve regurgitation is induced. This step leads to an increased amplitude of the systemic blood pressure and more pronounced reverse flow in the descending thoracic and abdominal aorta. The latter intervention allows a better closure of the donor graft after heterotopic implantation in the abdominal aorta.[1]

The second step involved the heterotopic, i.e. infrarenal implantation of a valve-bearing aortic graft from a donor animal. For all implantations in this experiment cryopreserved donor aortic grafts bearing the donor aortic valve were used. Donor grafts have been harvested 1-2 weeks prior to implantation. After explantation from donor animal, donor grafts were rinsed in saline and immersed in a cryopreservant solution before controlled freezing and storage at -80°C.
In this experiment, a cryopreserved graft was used because we intended to observe the effect of pioglitazone on the deterioration of the graft. In the herein applied model, cryopreserved grafts as well as other types of biological grafts, e.g. decellularized grafts, undergo a certain level of remodeling after implantation.[2]

Semi-quantitative assessment of graft calcification

The standardized assessment of clacification is an evaluation method that is applied on tissue sections subjected to von Kossa staining. From each explanted graft one tissue section is obtained for each of the four graft segments, i.e.: A1, A2, B1, B2 (Figure 2). Each graft segment is evaluated separately based on a well-defined scheme.
Tissue sections of A1 region (which corresponds to the level of the grafted aortic root including the grafted aortic valve) are scored by dividing each cross section into three parts: commissures, valve cusps, and annulus region. Each region is then evaluated based on the severity of observed calcification along pre-defined categories, resulting in 0-5 scoring points for each region.

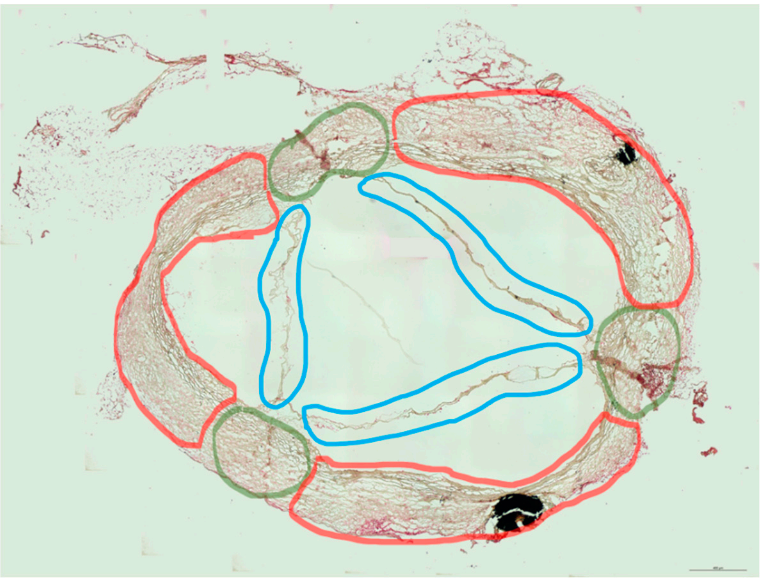

Red: Annulus
Blue: Leaflet
Green: Commissure

Tissue sections of the regions A2 to B2, i.e. tubular segments of the grafted aorta, are each divided into 8 sub-segments (also called zones), which are then also evaluated based on the severity of observed calcification and according to the same pre-defined categories, resulting

in 0-5 scoring points for each zone. The scoring points of each part are summed up to result in a total score result of a given graft region of a specific explant.

0 = no calcification
1 = microcalcification
2 = macrocalcification < 25% of the area
3 = macrocalcification 25% - 50% of the area
4 = macrocalcification 50% - 75% of the area
5 = macrocalcification > 75% of the area

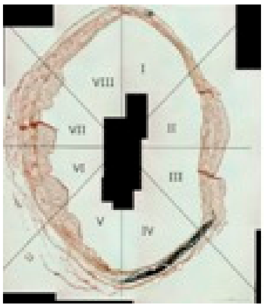

References:

[1] Munakata H, Assmann A, Poudel-Bochmann B, Horstkotter K, Kamiya H, Okita Y, Lichtenberg A, Akhyari P. *Aortic conduit valve model with controlled moderate aortic regurgitation in rats: a technical modification to improve short- and long-term outcome and to increase the functional results*. Circulation journal : official journal of the Japanese Circulation Society 2013;**77**:2295-302.
[2] Assmann A, Zwirnmann K, Heidelberg F, Schiffer F, Horstkotter K, Munakata H, Gremse F, Barth M, Lichtenberg A, Akhyari P. *The degeneration of biological cardiovascular prostheses under pro-calcific metabolic conditions in a small animal model*. Biomaterials 2014;**35**:7416-28.
