# Supplementary material for: PPAR-Gamma Activation May Inhibit the In Vivo Degeneration of Bioprosthetic Aortic and Aortic Valve Grafts under Diabetic Conditions
Source: Int J Mol Sci. 2021 Oct 14;22(20):11081. doi: 10.3390/ijms222011081 (PMC8538504; doi:10.3390/ijms222011081)
Supplement: Supplementary file 1 [file ijms-22-11081-s001.zip › Table S1_revised.pdf]

Table S1. Primers and respective sequences

Table S1. A list of the used primers and respective sequences

Gene	Forward primer (5’-3’)	Reverse primer (5’-3’)
TNF-alpha	GCTCCCTCTCATCAGTTCCA	GCTTGGTGGTTTGCTACGAC
IL-1b	AGGACCCAAGCACCTTCTTT	CATCATCCCACGAGTCACAG
IL-6	ACCACCCACAACAGACCAGT	AGTGCATCATCGCTGTTCAT
RAGE	TGAACTCACAGCCAATGTCC	TCAGAGGTTTCCAAG
BMP-2	GCTCAGCTTCCATCACGAA	AAGAAGAAGCGTCGGGAAGT
OCN	AAGCAGGAGGGCAGTAAGGT	GTCCGCTAGCTCGTCACAAT
OPN	AAGCCTGACCCATCTCAGAA	ATGGCTTTCATTGGAGTTGC
RUNX2	GATGACACTGCCACCTCTGA	GATGAAATGCCTGGGAACTG
ACTA2	GCTCTGGTGTGTGACAATGG	CCCCACATAGCTGTCCTTTT
OPG	GAGTGTGCGAATGTGAGGAA	CACCTGAGAAGAACCCATCC
MMP2	AGAAGGCTGTGTTCTTCGCA	GGTCAGTGGCTTGGGGTATC
MMP9	GCATCTGTATGGTCGTGGCT	TGCAGTGGGACACATAGTGG
MCP-1	CTGTAGCATCCACGTGCTGT	GGTGCTGAAGTCCTTAGGGT
NFκB	CCACTGTCAACAGATGGCCC	GCACACGTAGCGGAATCGAA

TNF-alpha, tumor necrosis factor-alpha; IL-1b, interleukin-1b; IL-6, interleukin-6; RAGE, receptor for advanced glycation end-products; BMP-2, bone morphogenetic protein-2; OCN, osteocalcin; OPN, osteopontin; RUNX2, runt-related transcription factor 2; ACTA2, actin alpha 2; OPG, osteoprotegerin; MMP2, matrix metalloproteinase 2; MMP9, matrix metalloproteinase 9; MCP-1, monocyte chemoattractant protein 1; NFκB, nuclear factor kappa-light-chain-enhancer of activated B cells
