# Supplementary figures and images for: PPAR-Gamma Activation May Inhibit the In Vivo Degeneration of Bioprosthetic Aortic and Aortic Valve Grafts under Diabetic Conditions
Source: Int J Mol Sci. 2021 Oct 14;22(20):11081. doi: 10.3390/ijms222011081 (PMC8538504; doi:10.3390/ijms222011081)

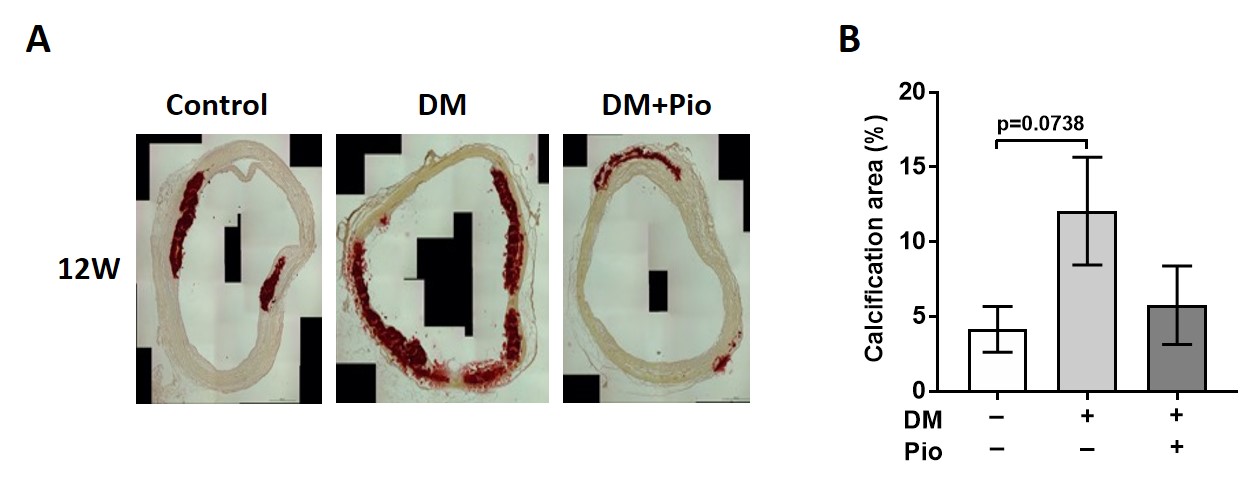

Supplement: Supplementary file 1 [file ijms-22-11081-s001.zip › Fig S1.png]

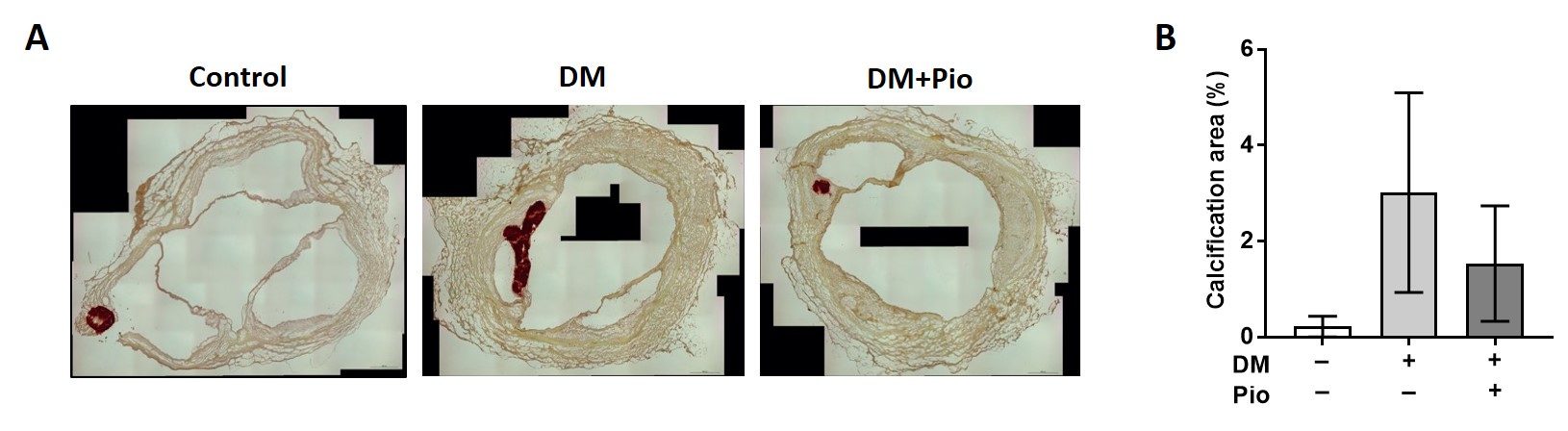

Supplement: Supplementary file 1 [file ijms-22-11081-s001.zip › Fig S2.png]

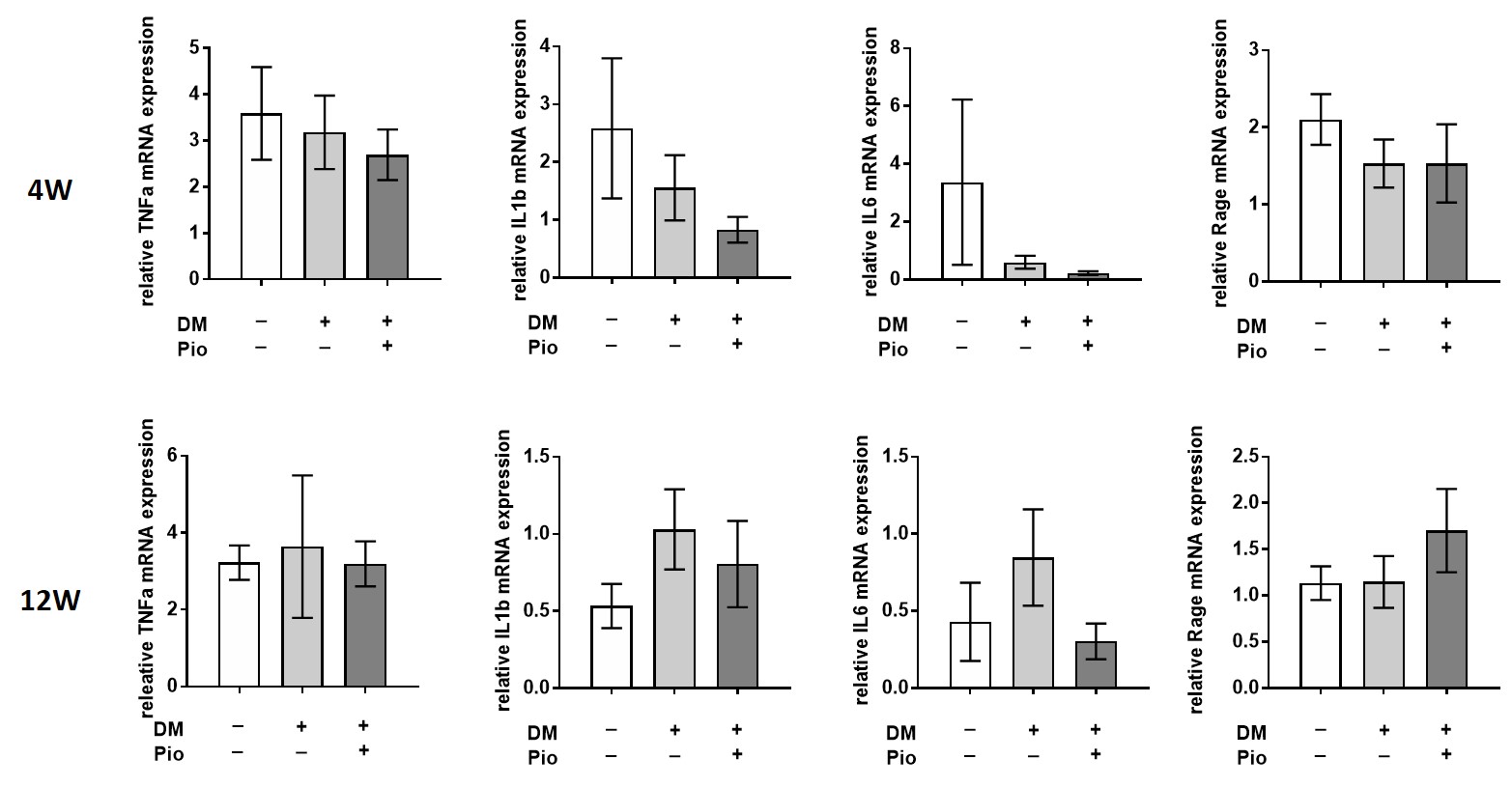

Supplement: Supplementary file 1 [file ijms-22-11081-s001.zip › Fig S3.png]

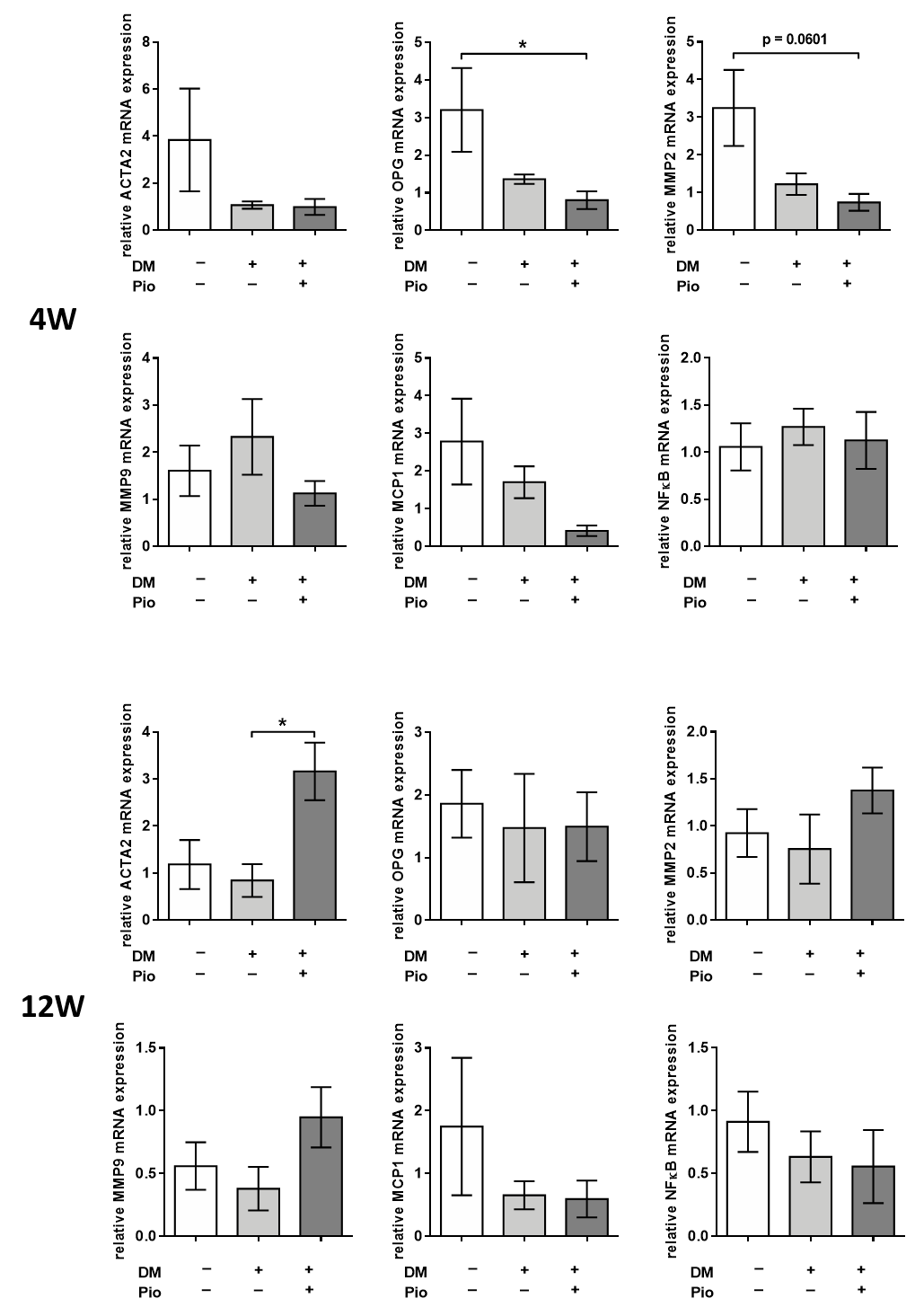

Supplement: Supplementary file 1 [file ijms-22-11081-s001.zip › Fig S4.tif]

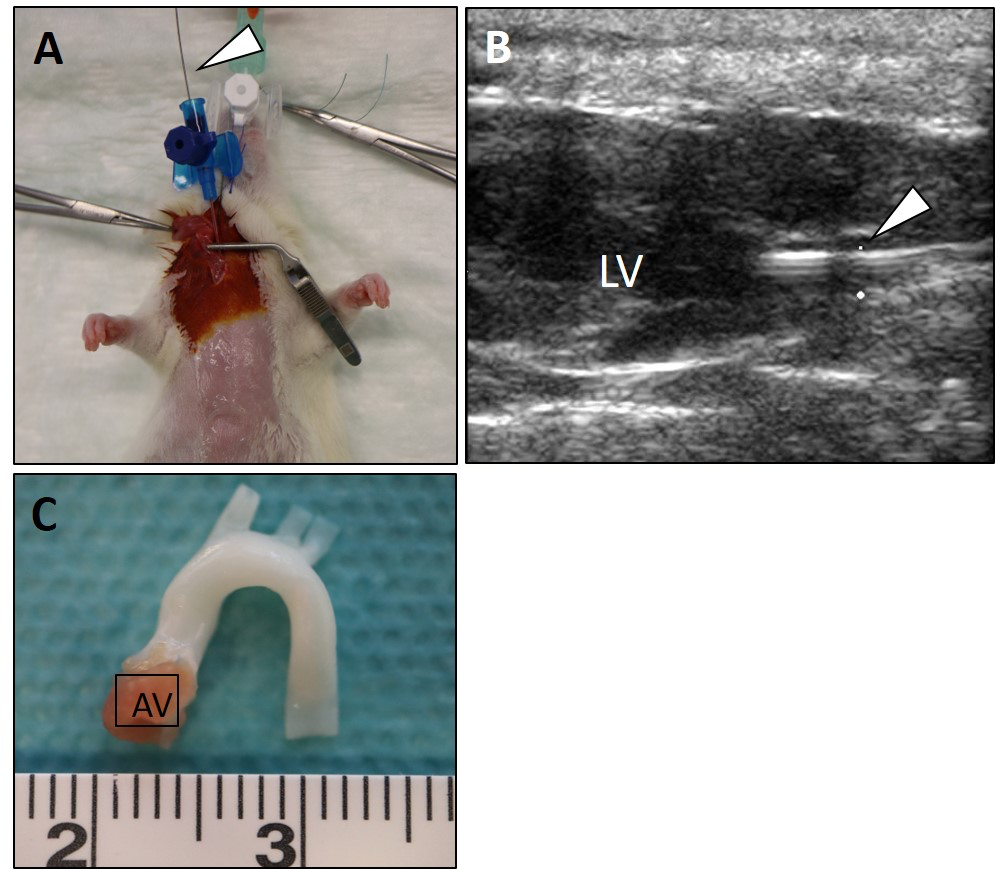

Supplement: Supplementary file 1 [file ijms-22-11081-s001.zip › Fig S5.png]
